# Diagnosis and management of subjects with mild cognitive impairment in clinical practice: an Italian delphi consensus study

**DOI:** 10.1007/s10072-026-09024-1

**Published:** 2026-04-20

**Authors:** Innocenzo Rainero, Emanuele Caggia, Bruno Brancasi, Claudia Carrarini, Guido Maria Giuffrè, Elisabetta Farina, Andrea Fabbo, Franco Giubilei, Laura Bonanni, Roberto Monastero, Claudio Paoli, Annachiara Cagnin

**Affiliations:** 1 Department of Neuroscience “Rita Levi Montalcini”, Memory Clinic, University of Torino, Via Cherasco, 15, Turin, 10126 Italy; 2 UOC Neurologia Ospedale di Caltagirone ASP 3 Catania, Caltagirone, Italy; 3 Department of Translational Biomedicine and Neuroscience “DiBraiN”, University of Bari “Aldo Moro”, Bari, Italy; 4 Neurology Unit, IRCCS San Raffaele Scientific Institute, Via Olgettina, 60, Milan, 20132 Italy; 5 Memory Clinic, Fondazione Policlinico Agostino Gemelli IRCCS, Rome, Italy; 6 Neurologia Riabilitativa e CDCD, IRCCS S.Maria Nascente, Fondazione Don C. Gnocchi, Milan, Italy; 7 Unità Operativa di Geriatria, Azienda ASL of Modena, Modena, Italy; 8 UOC Neurologia, Azienda Ospedaliero- Universitaria Sant’Andrea, Rome, Italy; 9 Department of Neurology, G. D’Annunzio University, Chieti, Italy; 10 Department of Biomedicine, Neuroscience and Advanced Diagnostic, University of Palermo, Palermo, Italy; 11 Department of Neurology, Livorno Hospital, Livorno, Italy; 12 Dipartimento di Neuroscienze and Padova Neuroscience Center, University of Padova, Padova, Italy

**Keywords:** Mild cognitive impairment, MCI, Management, Biomarkers, Acetylcholinesterase inhibitors, Citicoline

## Abstract

**Background:**

The prevalence of Mild Cognitive Impairment (MCI) is increasing worldwide, while the scarce evidence regarding clinical practice requires real-life research and consensus. This study examined real-life practices regarding the diagnosis and management of subjects with MCI in Italy.

**Methods:**

Data were collected from a modified Delphi study conducted in April-December 2023. The study involved 12 advisors and 16 specialists working in Italian Memory Clinics. The 50 statements proposed by the 12 advisors were rated on a 1–6 Likert scale in a two-round survey. Consensus was a priori defined when the rates of each statement in each round reached a mean value ≥ 5. The ratings of each statement were collected using an Excel spreadsheet. The data were analysed using Microsoft Excel and expressed as mean, median, and standard deviation (SD).

**Results:**

The response rate was 100% for both voting rounds. In the first round, 40 statements reached a consensus, while 8 did in the second. The SD of the eight final statements in the first round of voting ranged from 0.91 to 1.74, and that of the second round from 0.57 to 1.71.

**Conclusions:**

This consensus study emphasized the need to standardize investigations, set cut-off thresholds, and establish quantitative reporting standards. Building on this, the study recommended combining biomarker studies to stratify risks and predict decline. Furthermore, it advised integrating genetic data into risk algorithms and increasing testing and clinical criteria. Additionally, the consensus valued promoting specialized and integrative training, while fostering structured relationships with general practitioners.

## Introduction 

Over the past two decades, interest in studying and managing the prodromal phases of neurocognitive disorders has increased significantly. This enhancement has helped define preventive strategies that can reduce the risk of dementia and its progression. Mild cognitive impairment (MCI), a syndrome characterized by objective deterioration in cognitive functioning that does not compromise activities of daily living, has clearly emerged as a risk state for further cognitive and functional decline. MCI is a highly prevalent clinical syndrome: several international population-based studies estimated its prevalence between 15% and 20% in persons 60 years and older, with 5–15% of people developing dementia per year. However, approximately 50% remain stable at five years and, in a minority, symptoms resolve over time [1]. Given the heterogeneous presentations of MCI and the need for identifying criteria for differential diagnosis based on underlying aetiology, the syndrome was classified into different subtypes, such as MCI due to Alzheimer’s disease (AD) (NIA-AA criteria-2011), Vascular MCI (Am Heart Ass/Am Stroke Ass, 2011–2017), MCI due to dementia with Lewy Bodies [2], and MCI in Parkinson’s disease (2016). Cognitive testing, neuroimaging, and validated fluid biomarkers can improve the sensitivity and specificity of aetiological diagnosis of MCI. There is growing evidence that these tests may ease prognosis. Therefore, intercepting the prodromal onset, such as MCI or the preclinical phase, can allow interventions to delay or attenuate underlying neurodegenerative processes [3, 4]. All individuals with suspected MCI should undergo a comprehensive history and physical examination focusing on cognitive function, functional status, medications, neurological or psychiatric disturbances, and laboratory testing. These investigations are aimed at distinguishing MCI from normal aging or dementia and to identify potentially reversible forms of MCI due to other conditions, such as depression, thyroid diseases, and vitamin deficiency.

The international guidelines and consensus statements on the diagnosis and management of MCI [5, 6] have several discrepancies due to variations in the topics examined and the characteristics of different National Health Services. It is therefore essential to study and compare MCI recommendations, particularly in real-world settings. Given the complexity of the MCI condition and the limited evidence available in the literature, research and discussions on differential diagnosis tools, management, and treatments of MCI still leave open questions and require constant verification and consensus. This modified Delphi study evaluated the essential statements describing the current activities of Italian centres focussing on the uncertainty and essential needs posed by subjects with MCI in daily practice.

### Methods

The study was developed following the ACCORD guidelines for consensus methodology [7] applied to a modified Delphi study that utilized two rounds of qualitative surveys.

This study was not registered.

### Study design

Between April and December 2023, we conducted a Delphi study with an Italian panel of experts in MCI. Between April and May 2023, two advisory board meetings were held (in person and online). The two surveys were conducted on 30th June (first round) and 11th December 2023 (second round). The participants were asked to respond by email and phone calls by the study organizers (Summeet S.r.l.).

The experts’ panel was recruited from academic departments of psychiatry, cognitive/behavioural neurology, and geriatricians, medical associations or organizations, and was involved through formal invitations. The final panellists were experts in the clinical assessment of MCI.

The experts’ panel was composed of 12 advisors and 16 specialists. The panel consisted of 25 neurologists working in the most prominent Neurology centres specialized in dementia care in Italy, one psychiatrist, and two geriatricians. The macro-regional distribution of the panel was homogeneous: nine specialists in the North, nine in the centre, and ten in the Southern Italy.

The 12 advisors defined and authored the initial 51 statements based on a dedicated literature search and discussion to propose to the entire panel. The 12 advisors had voting rights, reviewed the results, and provided the manuscript.

The experts used a 1–6 Likert scale aimed to detecting agreement or disagreement. For each statement, the experts rated one to indicate total disagreement and six to indicate total agreement.

Consensus was a priori defined when the rates of each statement in every round achieved a mean value ≥ 5.

The panellists’ rates and comments were collected online by open-text documents regarding the ratings.

### Study procedure

The first-round survey utilized a questionnaire containing 51 statements. All the participants received the questionnaire by email and rated each statement independently. This survey aimed to explore the main approaches to managing the MCI in daily practice, as suggested by the advisory board experts.

The first question of the questionnaire concerned the participants’ personal details (their current position within the hospital and years of experience with MCI). Moreover, the questionnaire included a summary of the study, objectives, and an explanation of the procedure.

During the first-round survey, participants could add comments, proposals, suggestions, or modified the statements.

The results of the first-round survey (statements and rates) were provided to the panel members in a summary report. The statements obtained in the first-round survey were examined through a second-round survey performed by email. The objective of the second-round survey was to identify the essential approach modalities for managing subjects with MCI. The second-round survey yielded the final statements of this study.

The panel members were the same for both surveys. During both surveys, the immediate non-respondents were reminded by email or calls after 3–4 weeks. The missing answers were excluded from the analysis. The final consensus was established after the analysis of the second-round voting.

### Data collection and analysis

The ratings of each statement were collected using an Excel spreadsheet with the defined rating scale. The data were analysed using Microsoft Excel and expressed as mean, median, and standard deviation (SD).

Qualitative data and summarized statistics were reported in dedicated tables.

The study was not piloted and did not maintain anonymity.

## Results

For both rounds of voting, the number of complete answers was 28, corresponding to the number of panelists. Therefore, the response rates of both surveys were 100%.

## First-round survey

In the first-round survey performed on 30th June 2023, the 52 initial statements proposed to the panelists were rated. The first item regarded the panelists’ demographic data and professional titles; therefore, the statements to rate in the first-round survey were 51. Table 1 reports the 51 statements used in the first-round survey alongside the relative rates and the statements that subsequently achieved consensus through the second-round survey (highlighted in grey).

**Table 1:** Statements rated at first survey

Delphi code	Statement	First round rates		
		Mean	Median	SD
2	MoCA should be considered the gold standard for the cognitive screening of MCI, and it is superior to MMSE for the timely diagnosis of this condition.	4.57	5.00	157
3	The neuropsychological assessment of the second level is the gold standard for the MCI diagnosis.	5.54	6.00	0.82
4	The GP (General Practitioner) plays a crucial role in the screening pathway of MCI to intercept the disease and reduce underdiagnoses.	5.50	6.00	0.82
5	The GPCOG is a reliable and easy-to-use tool for the MCI diagnosis by GPs.	4.50	5.00	0.91
6	It is appropriate to encompass the Hachinski scale in the ordinary diagnostic work-up of MCI.	3.61	3.00	1.47
7	It is appropriate to include a depression scale in the ordinary diagnostic work-up of MCI.	5.29	6.00	0.88
8	It is appropriate to include in the ordinary diagnostic work-up of MCI a correct pharmacological anamnesis about drugs that can be confounding factors.	5.82	6.00	0.47
9	The multi-domain neuropsychological assessment for MCI should evaluate at least five cognitive domains: memory, attention/concentration, executive and visuospatial functions, and language.	5.79	6.00	0.62
10	The neuropsychiatric inventory (NPI) should be administered to all patients with MCI.	4.61	5.00	1.74
11	The presence of a reliable caregiver is essential during the first visit of an MCI patient.	5.64	6.00	0.97
12	The classification of amnestic or non-amnestic MCI is helpful for diagnostic and prognostic purposes.	5.39	6.00	0.94
13	The classification of MCI in single or multiple domains is helpful for prognosis.	5.43	6.00	0.90
14	A timely diagnosis of MCI allows the patient and family to modify their lifestyle and optimize the therapy.	5.86	6.00	0.35
15	The FCSRT is the gold standard for assessing episodic memory impairments of hippocampal type in subjects with MCI.	5.00	5.00	0.96
16	The subjective cognitive disorder has to be investigated and controlled over time as it has a clinical prognostic value for MCI and dementia.	5.46	6.00	0.82
17	The diagnostic work-up for MCI should include a screening test for apathy.	4.57	5.00	1.32
18	It is appropriate to guarantee a minimum number of CDCDs at a regional and local level for correctly managing the diagnostic pathways and clinical care management.	5.93	6.00	0.26
19	In every CDCD, it would be advisable that a medical professional with neurological, but also geriatric and psychiatric skills is present.	5.64	6.00	0.72
20	It is appropriate to advise against driving the subjects with MCI.	2.89	3.00	1.42
21	Subjects with MCI should be proposed a therapy with acetylcholinesterase inhibitors, memantine, or both.	3.54	4.00	1.61
22	For subjects with MCI, an individual or group cognitive stimulation pathway or occupational therapy would be suggestable.	5.00	5.00	1.13
23	Planning a psychological support service in the CDCD dedicated to patients and caregivers is opportune.	5.79	6.00	0.41
24	Telemedicine can be a helpful tool for managing patients with cognitive deterioration to monitor their clinical progress.	5.14	6.00	1.03
25	Beyond diagnostic tools, the CDCD should provide informative, educational, training, and relational tools to patients, their family assistants, and caregivers.	5.79	6.00	0.49
26	To start prevention measures, strategies and possible therapies for cognitive deterioration or impairment progression must be immediate.	5.89	6.00	0.31
27	The MCI diagnosis must be as early as possible but after a suitable in-depth analysis to lessen the overdiagnosis risk.	5.68	6.00	0.54
28	The onset of psycho-behavioural or cognitive disorders beyond 50 years of age and in the absence of other organic or iatrogenic causes should raise the suspicion of a neurodegenerative process.	5.50	6.00	0.63
29	It is essential to evaluate the comorbidities of the patients with cognitive deterioration or impairment to improve the prognosis and optimize the therapy.	5.82	6.00	0.47
30	The early diagnosis of MCI and the treatment introduction have a whole economic positive and social direct and indirect value.	5.46	6.00	0.68
31	The research should propose suitable tools for early diagnosis for a better local and territorial management of MCI.	5.71	6.00	0.52
32	Mild behavioural impairment (MBI) is a late-onset neurobehavioral syndrome that can surge at any point of the pre-dementia spectrum, from the conditions of cognitive normality to the subjective cognitive or mild cognitive deficit, and should be assessed with ad hoc tools (e.g., Mild Behavioural Impairment Checklist).	5.46	6.00	0.78
33	It is necessary to promote integrated relationships among specialist centres and territorial medicine for the early diagnosis, follow-up, and management of patients with MCI.	5.79	6.00	0.41
34	The encephalic MRI is the neuroradiological examination of first choice (except for contraindications) for patients with MCI, even for evaluating differential diagnosis.	5.50	6.00	0.94
35	It is helpful to complete structural imaging examinations with specific indexes of global and focal atrophy and on the burden of vascular (ischemic and micro haemorrhagic) damage.	5.64	6.00	0.61
36	In the MCI, functional neuroimaging can help as a second-level investigation and should be supported by specific appropriateness criteria.	5.43	6.00	0.90
37	In patients with persistent or progressive MCI, it is appropriate to analyse the cerebrospinal liquid or perform cerebral PET with the radiotracer for amyloid.	4.93	5.00	1.13
38	In pre-senile subjects or when there are diagnostic doubts, it is appropriate to analyse the cerebrospinal liquid or perform cerebral PET with the radiotracer for amyloid to diagnose Alzheimer’s disease.	5.50	6.00	1.09
39	Plasma biomarkers of neurodegeneration and neuro-inflammation can help diagnose patients with MCI and identify the disease progression.	4.82	5.00	1.31
40	Evaluating the APOE gene polymorphisms helps diagnose the mild cognitive or subjective cognitive deficit.	4.07	4.00	1.62
41	An in-depth laboratory analysis (folates, B12 vitamin, thyroid functionality) is appropriate to exclude possible secondary causes of cognitive deterioration.	5.79	6.00	0.49
42	The timely differential diagnosis of MCI can allow the interested subjects to participate in clinical trials.	5.71	6.00	0.65
43	In patients with MCI, all the cardiovascular risk factors must be strictly controlled.	5.64	6.00	1.01
44	The mood disorders must be treated appropriately in every stage of the neurocognitive disorder.	5.64	6.00	0.77
45	It is appropriate to advise patients with MCI on possible experimental therapies, if available.	5.39	6.00	0.90
46	The treatment of patients with MCI with choline donors may have positive effects on cognitive functions.	5.11	5.00	0.94
47	The treatment of patients with MCI with nutritional integrators can have positive effects on cognitive functions	5.18	5.00	0.76
48	Adequate therapy of minor or major humour disorders improves the prognosis of patients with MCI.	5.32	5.50	0.80
49	The new etiopathogenetic therapies for Alzheimer’s disease must be first reserved for well-defined cases according to inclusion and exclusion criteria to define their validity and limitations, thus indications.	5.54	6.00	1.24
50	It is always appropriate to prescribe to patients with MCI well being lyfestile and dietetic measures adequate to slow the disease progression and the evolution toward the major neurocognitive disorder.	5.61	6.00	0.62
51	Patients with MCI may benefit from the treatment of possible sleep disorders.	5.86	6.00	0.35
CDCD, Centri per i Disturbi Cognitivi e Demenze; FCSRT, Free and Cued Selective Reminding Test; GPCOG, General Practitioner Assessment of Cognition; MBI, Mild Behavioural Impairment; MMSE, Mini Mental State examination; MoCA, Montreal Cognitive Assessment; MCI, Mild Cognitive Impairment; MRI, Magnetic Resonance Imaging; NPI, Neuropsychiatry Inventory; PET, positron emission tomography.				

### Second-round survey

In the first-round survey, 40 statements reached a mean value rating of 5 or higher. These 40 statements were reassessed in the second-round survey of 4th December 2023. The rating of the second-round survey yielded eight final statements that reached a final consensus reported in Table 2, which also includes three statements that achieved a mean value ≥ 5 in the first but not at the second round. The Delphi codes of these eight final statements were: 2, 10, 17, 37, 39, 43, 20, and 46. No additional statement was introduced during the two rounds of surveys. However, three other statements (Delphi codes: 5, 40, and 21) did not achieve a mean rating of 5 or higher in the second-round survey, but they achieved a mean rating of 5 during the first-round survey, as reported in Table 2.

**Table 2:** The final statements achieved at the second round

Delphi code	Statements	First round rates			Second round rates			Percentage of panellists who rated each statement ≥ 5	
		Mean	Median	SD	Mean	Median	SD		
2	MoCA (Montreal Cognitive Assessment) is superior to MMSE (Mini Mental State Examination) in terms of sensibility and specificity for the timely diagnosis of this condition.	4.57	5.00	1.57	5.00	6.00	1.58	66.67	
5*	The GPCOG (General Practitioner Assessment of Cognition) scale is a sensible tool, reliable, and easy to use for assessing the initial cognitive deficit by General Practitioners.	4.5	5.00	0.91	4.83	5.00	1.14	33.33	
10 Different wording	It is suggestible to administer the neuropsychiatric inventory (NPI) to patients with MCI who show behavioral disorders.	4.61	5.00	1.74	5.46	6.00	0.96	66.67	
17 Different wording	A test for apathy should be considered for the diagnostic in-depth analysis for MCI in selected cases.	4.57	5.00	1.32	5.50	6.00	0.65	58.33	
37 Different wording	In the cases with persistent and progressive MCI, it is indicated to discuss with the patients the possibility of performing the cerebrospinal liquid analysis or the cerebral PET with radiotracers for amyloid.	4-0.93.93	5.00	1.13	5.46	6.00	0.87	70.83	
39 Different wording	In the future, plasmatic biomarkers of neurodegeneration and neuroinflammation will be helpful for the diagnosis of patients with MCI and for identifying the disease progression.	4.82	5.00	1.31	5.54	6.00	0.76	70.83	
40* Different wording	The assessment of the APOE gene polymorphisms is helpful for the diagnostic evaluation and determining the evolution risk in the subjects with MCI and the subjective cognitive deficit.	4.07	4.00	1.62	4.71	5.00	1.06	29.17	
43 Different wording	It is appropriate to include the screening of the cardiovascular risk factors in the ordinary diagnostic work-up for MCI.	5.64	6.00	1.01	5.58	6.00	0.57	62.50	
20 Different wording	It is appropriate to discuss with patients with MCI and their family members about the possible risks associated with driving and to suggest caution strategies.	2.89	3.00	1.42	5.29	6.00	1.02	58.33	
46 Different wording (previous choline donors)	Treating of the MCI condition with citicoline can be helpful for the possible positive effects on cognitive functions.	5.11	5.00	0.94	5.29	6.00	0.93	58.33	
21* Different wording	For subjects with MCI, a therapy with acetylcholinesterase inhibitors can be proposed according to the specialist’s opinion.	3.54	4.00	1.61	4.46	5.00	1.71	33.33	
*statements that achieved a mean value ≥ 5 at the first round but not at the second.									
CDCD, Centri per i Disturbi Cognitivi e Demenze; FCSRT, Free and Cued Selective Reminding Test; GPCOG, General Practitioner Assessment of Cognition; MBI, Mild Behavioural Impairment; MMSE, Mini Mental State examination; MoCA, Montreal Cognitive Assessment; MCI, Mild Cognitive Impairment; MRI, Magnetic Resonance Imaging; NPI, Neuropsychiatry Inventory; PET, positron emission tomography.									

Seven out of the final eight items (Delphi codes: 10, 17, 37, 39, 43, 20, and 46) expressed statements with wording different from those initially proposed in the first list of 50. For example, the statement 37 about the analysis of cerebrospinal fluid or cerebral PET with radiotracers for amyloid was changed from “advisable” to “indicated” [AGG] to reinforce the importance of this investigation [RM] (Table 3).

**Table 3:** The final statements achieved at the second round versus the Italian National Institute of Health guidelines

Delphi code	Statements	
2	MoCA (Montreal Cognitive Assessment) is superior to MMSE (Mini Mental State Examination) in terms of sensibility and specificity for the timely diagnosis of this condition.	In line
10 Different wording	It is suggestible to administer the neuropsychiatric inventory (NPI) to patients with MCI who show behavioral disorders.	More permissive
17 Different wording	A test for apathy should be considered for the diagnostic in-depth analysis for MCI in selected cases.	More permissive
37 Different wording	In the cases with persistent and progressive MCI, it is indicated to discuss with the patients the possibility of performing the cerebrospinal liquid analysis or the cerebral PET with radiotracers for amyloid.	More permissive
39 Different wording	In the future, plasmatic biomarkers of neurodegeneration and neuroinflammation will be helpful for the diagnosis of patients with MCI and for identifying the disease progression.	More permissive
43 Different wording	It is appropriate to include the screening of the cardiovascular risk factors in the ordinary diagnostic work-up for MCI.	In line
20 Different wording	It is appropriate to discuss with patients with MCI and their family members about the possible risks associated with driving and to suggest caution strategies.	More restrictive
46 Different wording (previous choline donors)	Treating of the MCI condition with citicoline can be helpful for the possible positive effects on cognitive functions.	More permissive

CDCD, Centri per i Disturbi Cognitivi e Demenze; FCSRT, Free and Cued Selective Reminding Test; GPCOG, General Practitioner Assessment of Cognition; MBI, Mild Behavioural Impairment; MMSE, Mini Mental State examination; MoCA, Montreal Cognitive Assessment; MCI, Mild Cognitive Impairment; MRI, Magnetic Resonance Imaging; NPI, Neuropsychiatry Inventory; PET, positron emission tomography

The SD of the final eight statements obtained at the first-round survey varied from 0.91 to 1.74 and of the second-round survey from 0.57 to 1.71.

**Fig. 1:**
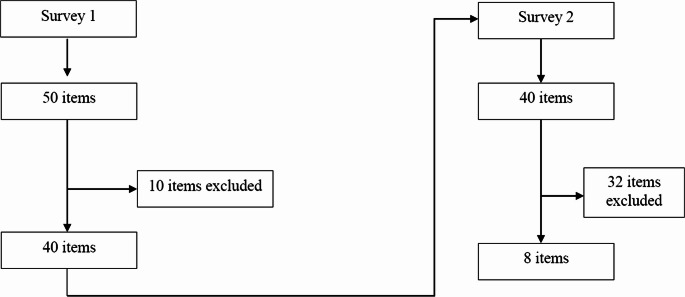
Flow chart representing the modified Delphi process used in the present study

Figure 1 shows the whole process of the modified Delphi method applied in this study.

## Discussion

This consensus study focused on real-world data collected through a survey of Italian specialists working in Memory Clinics located in the North, Centre, and Southern Italy, for the diagnosis and treatment of subjects with MCI. The collected data were mainly compared with the recent guidelines for MCI provided by the Italian National Institute of Health. Following table must be moved after Conclusions

**Table:** 

MCI: practical suggestions	
MoCA	For timely diagnosis
NPI	For patients with behavioral disorders
Apathy test	For in.depth analysis of MCI (selected cases)
Cerebrospinal liquid analysis/cerebral PET	For persistent and progressive MCI
Screening of cerebrovascular risk factors	For ordinary diagnostic work-up
Caution strategies	For risks associated with driving
Citicoline treatment	For possible positive effects on cognitive function
Plasmatic biomarkers	For diagnosis and prognosis of disease progression

### Assessment: neuropsychological evaluation and biomarkers

After excluding concomitant diseases, the initial screening of subjects with MCI requires an appropriate neuropsychological evaluation. The guidelines of the Italian National Institute of Health provided a strong positive recommendation about the neuropsychological assessment of subjects with MCI (Recommendation n° 28). This initial screening includes validated tests of episodic memory as an integral part of the diagnostic pathway of MCI and its subtypes in a specialist setting [8]. Among the assessment neuropsychological tools, in the second-round survey of our study, the panellists reached a consensus about the preferred use of Montreal Cognitive Assessment (MoCA) (Delphi code n° 2) over MMSE [9]. The Manchester consensus recommended using MoCA as the cognitive screening tool for MCI diagnosis, with a 25/25 cut-off point. The sensitivity of the MoCA test was 80.48% and the specificity was 81.19% [10]. However, the diagnosis of MCI based only on the MoCA to assess specific cognitive domains was deemed insufficient by several researchers [4, 11].

Additionally, the General Practitioner Assessment of Cognition (GPcog) test received a strong positive recommendation from the guidelines of the Italian National Institute of Health (Recommendation n°3) as it is the only cognitive test validated for initial assessment in primary care in the Italian population. In our study, the statement regarding the GPcog (Delphi code n° 5) did not reach sufficient consensus with a mean value of 4.5 in the first-round survey and 4.83 in the second-round survey. Less stringent statements might have achieved more consensus, such as “The GPcog is a useful tool for assessing initial cognitive impairment by GPs”; however, the GPcog scale is not listed among the cognitive tests in a non-specialist setting by Italian National Institute of Health (Recommendation n. 3) [8].

During the second-round survey, panelists excluded the statement concerning the Free and Cued Selective Reminding Test (FCSRT) as a diagnostic tool for the diagnosis of MCI. The FCSRT assesses verbal episodic memory through controlled learning and semantic cueing, and is recommended for identifying encoding and storage deficits characterizing AD-related memory disorders [12]. While the FCSRT can play a crucial role in detecting prodromal phases of AD, its utility is limited in non-AD-related pathologies.

Clinicians should assess subjects with MCI for behavioral and neuropsychiatric symptoms, as these symptoms may indicate a high likelihood of conversion from any MCI type to dementia. In the present study, the assessment of subjects with MCI using the Neuropsychiatry Inventory (NPI) test (Delphi code n° 10) achieved a consensus in the first round, suggesting a substantial agreement on the usefulness of the test. A specific statement regarding testing apathy (Delphi code n° 17) achieved a consensus in the second-round survey, with a mean value of 5.5. To note, the guidelines of the Italian National Institute of Health did not provide specific recommendations for testing apathy [8]. Apathy is a highly prevalent symptom in subjects with neurocognitive disorders and is associated with faster cognitive and functional decline, decreased quality of life, and increased mortality [13]. Furthermore, apathy must not be confused with depression or anxiety; thus, the use of specific tests for the investigation of apathy in subjects with MCI needs further attention.

Supporting the neuropsychological diagnosis of MCI with biomarkers is crucial, yet it remains a topic of debate as no validated biomarker is currently available for diagnostic purposes or to differentiate the MCI subtypes [14]. In our study, the statement regarding the need to detect beta-amyloid pathology in cerebral fluid or PET imaging reached a consensus at the second-round survey, with a mean value of 5.46, in case of persistent or progressive MCI (Delphi code n°37). However, the guidelines of the Italian National Institute of Health do not currently include recommendations for investigating beta-amyloid pathology, particularly as prodrome of AD [8]. Moreover, as MCI and cognitive decline may progress even in the absence of beta -amyloid pathology, other hallmarks should be identified to characterize individuals whose condition evolves over time [15].

The assessment of ApoE gene polymorphisms (Statement Delphi code 40) did not reach a consensus among our panelists and received a strong negative recommendation from the Italian National Institute of Health (Recommendation 17). The statement might have reached a consensus if was phrased as “The evaluation of the polymorphisms of the ApoE gene can be helpful for the prognostic assessments relative to the risk of evolution in subjects with MCI” or “The evaluation of the ApoE polymorphisms, whereby possible, provides prognostic information about the likelihood of progression to dementia in subjects with MCI”.

Among the neuroimaging (mainly MRI) investigations, one of the neurobiological markers of MCI intermediate stage is focal atrophy [4, 11]. In the first-round survey of the present study, the statement Delphi code n° 35 (“It is helpful to complete structural neuroimaging examinations with specific indexes of global and focal atrophy and on the burden of vascular (ischemic and micro hemorrhagic) damage”) achieved a mean rate of 5.64; however, this statement was excluded during the second-round process of our study. The guidelines of the Italian National Institute of Health do not currently include recommendations about the diagnostic role of investigating brain atrophy patterns. Indeed, several approaches applied for subtyping subjects with MCI according to brain atrophy patterns are currently under development to provide insights alongside neuropsychological tests [16].

Plasma biomarker tests for a differential diagnosis had a new strong negative recommendation by the Italian National Institute of Health (Recommendation n°29) [8]. In our study, the statement about the use of biomarkers for MCI as future tools (Delphi code n° 39) reached a consensus, yet in the second-round survey. In 2020, an Italian consensus by a task force focused on identifying biomarkers appropriate to an etiological diagnosis, but did not provide defined guidelines due to limited evidence [17]. The preparatory phase of a European consensus about the MCI diagnosis based on rational use of biomarkers was established with the aim of stratifying the risk factors of dementia progression and a tailored workflow on the patients’ profiles [5, 18].

The future standard assessment of MCI should consider advancements in neurobiological research [4]. Also, the etiological diagnosis of MCI should be based on a combination of biomarkers, including clinical and cognitive evaluations, fluid analysis, and neuroimaging investigations. This comprehensive approach will help address the etiological complexity associated with MCI.

### Work-up and management

Concerning comorbidities, the screening for risk of cardiovascular diseases (Delphi code n° 43), such as hypertension [19, 20], was considered part of the usual work-up of MCI management by our panel and reached the consensus in both the rounds of this study, aligned with the strong recommendation of the Italian National Institute of Health about the treatment of cardiovascular diseases and other comorbidities (Recommendation n° 69) [8].

The statement about driving vehicles (Delphi code n° 20) reached a consensus in the second-round survey of our study, with a mean value of 5.29. The guidelines of the Italian National Institute of Health do not provide any specific recommendation about driving safety; however, monitoring driving capabilities has been proposed as a research questions (Question n° 8b) [8].

Our experts did not reach a consensus on the value of the support provided by the Centers for Cognitive Disorders and Dementia (CDCDs), even though their use is strongly recommended by the Italian National Institute of Health for non-specialist setting (Recommendation n° 5) [8]. Subjects with MCI may be diagnosed and followed mainly by general practitioners. In the event of cognitive progression, the subjects with MCI should be followed by the network of CDCDs for further investigations.

### Treatments

In the present study, pharmacological treatment of MCI with acetylcholinesterase inhibitors (Delphi code 21) was excluded in accordance with the strong negative recommendation provided by the Italian National Institute of Health [8, 14]. However, several randomized controlled trials and observational studies investigated the safety and efficacy of cholinesterase inhibitors and memantine in subjects with MCI. In terms of efficacy, a meta-analysis has shown that these drugs could significantly improve the MMSE and MoCA scores, but they are not able to significantly reduce ADAS-cog scores or delay the disease progression. In terms of safety, cholinesterase inhibitors could significantly increase the risk of adverse reactions, such as nausea, vomiting, and diarrhoea [21]. However, there is growing interest in evaluating the efficacy and safety of cholinesterase inhibitors in MCI subtypes (e.g., MCI due to AD) defined using biomarkers. However, this strategy requires further studies. In our study, treatment of subjects with MCI with citicoline and choline precursors achieved consensus at first and second-round surveys, given the possible positive effects on cognitive functions [8]. Among the cholinergic medications, citicoline has been proposed as providing neuroprotective effects through diverse mechanisms of action. Treatment with citicoline led to a consistent improvement in cognitive function in subjects with MCI, especially those of vascular origin [22]. The long-term treatment with citicoline proved well-tolerated and not associated with severe adverse events [22]. Moreover, the treatment with citicoline in combination with acetylcholinesterase inhibitors and/or memantine in patients with mixed dementia and AD showed effectiveness, with an increased MMSE score over time [23].

### Limitations

The first limitation of the present study is the relatively long time (approximately six months) between the two rounds of surveys. Moreover, the number of panelists may have been insufficient to evaluate the complex strategies needed for the diagnosis and treatment of MCI. However, the specialists involved in the consensus study represented CDCDs from all Italian regions.

Some statements eliminated during the process obtained a mean value equal to or greater than 5 in the first-round survey, but not in the second. The wording of some final statements was different from the corresponding initial list. These text changes were made to clarify or refine the concepts expressed in the initial development of the statements. However, the differences in wording between the first and second-round surveys may have influenced the rates of the panelists.

Finally, the lack of anonymity, which was due to the small number of panelists, may have introduced a possible dominance bias.

In summary, the pattern emerged from this consensus study can be delineated as:

- confirmed that the diagnosis of MCI is challenging for specialists, requires a personalized approach for each patient, and necessitates the utmost attention and highlighted that the MCI diagnosis and evaluation scales may have a low level of uniformity;
- underscored the need for an optimal combination of clinical, neuroimaging, neuropsychological, and biomarker evaluations to contribute to differential diagnosis and prognosis of MCI;
- highlighted the utmost importance of biomarkers, once their validation is established;
- verified that the current management remains primarily focused on the assessment tools (mainly neuropsychological in progressive cases and neuroimaging investigation);
- acknowledged the strategic importance of assessment of comorbidities (especially cardiovascular) for possible reversion of MCI after the removal of temporary causes of MCI;
- paid particular attention to the selection of patient profiles to provide personalized pharmacological treatments and non-pharmacological interventions for lifestyle changes (e.g., driving safety) that involve proactive communication on the part of family members/caregivers;
- confirmed that pharmacological treatment of MCI is still a matter of debate. The main reason for such a problem is that MCI is a syndromic diagnosis, and only the use of biomarkers may specify the aetiology of such a syndrome.

## Conclusions

The results of this consensus study highlighted some particular aspects of the complex approach required by MCI in Italy. To optimize the diagnosis, management, and treatment of MCI, it is necessary to standardize investigations, cut-off thresholds, and quantitative reports; combine biomarkers studies to stratify risk and predict the course; integrate the genetic data into the risk algorithm; increase the acceptability of the instrumental investigations; educate the staff involved in the use of registries, test and clinical criteria; promote a specialist and integrative training and structured relationship with the general practitioners. To achieve these goals leading to improved clinical research on MCI, it is essential to share real-world experiences and monitor the evolution of evidence over time.

## Data Availability

Data achieved during the current study are available from the Corresponding author [Rainero Innocenzo] upon reasonable request.

## Abbreviations

ABC: Anticholinergic Cognitive Burden Scale
AD: Alzheimer’s disease
CDCD: Centri per i Disturbi Cognitivi e Demenze
DT: Digital Technologies
FCSRT: Free and Cued Selective Reminding Test
GP: General Practitioner
GPCOG: General Practitioner Assessment of Cognition
MBI: Mild Behaviour Impairment
MCI: Mild Cognitive Impairment
MMSE: Mini Mental State Examination
MoCA: Montreal Cognitive Assessment
MRI: Magnetic Resonance Imaging
NPI: Neuropsychiatric Inventory
PET/CT: Positron Emission Tomography/Computed Tomography
UVA: Unità Valutazione Alzheimer
